# Phylogenetic Structure Revealed through Combining DNA Barcodes with Multi-Gene Data for *Agrodiaetus* Blue Butterflies (Lepidoptera, Lycaenidae)

**DOI:** 10.3390/insects14090769

**Published:** 2023-09-15

**Authors:** Vladimir A. Lukhtanov, Nazar A. Shapoval, Alexander V. Dantchenko, Wolfgang Eckweiler

**Affiliations:** 1 Department of Karyosystematics, Zoological Institute, Russian Academy of Sciences, Universitetskaya Nab. 1, 199034 Saint-Petersburg, Russia; alex.danchenko@gmail.com; 2 Gronauer Street 40, D-60385 Frankfurt, Germany; b2010@lycaena.de

**Keywords:** biodiversity, *COI*, DNA barcoding, insects, Lepidoptera, *Polyommatus*, taxonomy

## Abstract

**Simple Summary:**

The species-rich subgenus *Agrodiaetus* Hübner, 1822 is a distinct monophyletic lineage within the diverse blue butterfly genus *Polyommatus* Latreille, 1804. Although the subgenus has attracted the attention of numerous researchers, a large number of unresolved taxonomic problems persist in *Agrodiaetus*. In our study, we analyzed the taxonomic structure of the subgenus via combining short mitochondrial DNA barcodes of several extremely rare species, for which multi-locus data were unavailable, with multi-gene mitochondrial and nuclear data for the other *Agrodiaetus* taxa. This approach resulted in a high phylogenetic resolution of the tree obtained, even for the clades, which were solely represented by DNA barcodes. The status and taxonomic position of the enigmatic species *P. muellerae*, *P. afghanicus*, *P. frauvartianae*, *P. bogra* and *P. anticarmon* from Afghanistan, Pakistan, Iran and Turkey are revealed and discussed. The complete list of species groups and species of the subgenus *Agrodiaetus* is presented.

**Abstract:**

The need for multi-gene analysis in evolutionary and taxonomic studies is generally accepted. However, the sequencing of multiple genes is not always possible. For various reasons, short mitochondrial DNA barcodes are the only source of molecular information for some species in many genera, although multi-locus data are available for other species of the same genera. In particular, such situation exists in the species-rich butterfly subgenus *Polyommatus* (*Agrodiaetus*). Here, we analyzed the partitioning of this subgenus into species groups by using three data sets. The first data set was represented by short mitochondrial DNA barcodes for all analyzed samples. The second and third data sets were represented by a combination of short mitochondrial DNA barcodes for part of the taxa with longer mitochondrial sequences *COI* + *tRNA-Leu* + *COII* (data set 2) and with longer mitochondrial *COI* + *tRNA-Leu* + *COII* and nuclear *5.8S rDNA* + *ITS2* + *28S rDNA* sequences (data set 3) for the remaining species. We showed that the DNA barcoding approach (data set 1) failed to reveal the phylogenetic structure, resulting in numerous polytomies in the tree obtained. Combined analysis of the mitochondrial and nuclear sequences (data sets 2 and 3) revealed the species groups and the position within these species groups, even for the taxa for which only short DNA barcodes were available.

## 1. Introduction

Ideally, the analysis of evolutionary history and taxonomic structure of living organisms requires a comparative analysis of data obtained from multiple sources of evidence (morphological, multi-locus molecular, ecological, karyological, etc.) [1,2,3,4,5]. In practice, such comprehensive analysis is not always possible. Many species are extremely rare and represented in collections by a limited number of specimens. Usually, such museum material is hardly suitable for comprehensive multi-locus molecular analysis due to its old age—resulting in DNA degradation—and the view that unique samples (especially type specimens) should be preserved as important standard vouchers rather than destroyed in the course of molecular studies.

In this situation, massive single-locus sequencing studies, such as the DNA barcoding research presented in Refs [6,7], have become the only real means of obtaining regular molecular information, which is available for multi-species comparisons and can thus be incorporated into phylogenetic research and taxonomic revisions. A situation where, for some species of a genus, there are only mitochondrial DNA barcodes, and for other species of the same genus, there are multi-gene data, is ordinary [4]. Recently, a novel approach has been suggested for phylogenetic analysis of such genera [8]. This approach is based on the combined analysis of short mitochondrial DNA barcodes for all species of a genus with multi-locus data for several representative taxa of the same genus.

Mitochondrial DNA barcodes, as well as their combination with nuclear genes, have been widely used to solve taxonomic problems of varying complexity, e.g., in butterflies (Refs [9,10,11,12,13,14]). An increase in the length of the molecular matrix up to the phylogenomic and genome-wide data dramatically increases the resolution of phylogenetic and taxonomic analyses [15]. However, combining short sequences for some species with long sequences for other species can theoretically be problematic because missing characters can negatively affect the topology and branch length estimation [16]. A study by Talavera et al. [8] clearly demonstrated that increasing species sampling by adding short DNA barcodes to multi-locus matrices increases the phylogenetic resolution. This is probably due to a partial solution to the problems of incomplete species sampling and long branch attraction [8].

In our study, we applied this approach [8] to the analysis of phylogenetic structure in the species-rich butterfly subgenus *Polyommatus* (*Agrodiaetus*) Hübner, 1822 (Lepidoptera, Lycaenidae). This subgenus represents a distinct monophyletic lineage within the diverse genus *Polyommatus* Latreille, 1804 [4]. The subgenus *Agrodiaetus* was estimated to have originated only about three million years ago [17] and radiated rapidly in the Western Palaearctic region [18]. The last published review of the subgenus includes 120 valid species [19]. Species within *Agrodiaetus* are extremely uniform and, with a few exceptions, exhibit few differences in characteristics traditionally used in classification, such as wing pattern and/or aspects of the male and female genitalia [17]. At the same time, these species vary greatly in their karyotypes, with the haploid chromosome numbers (n) ranging from n = 10 to n = 134 [18]. Therefore, it is not surprising that most of the work on the taxonomy of the subgenus is based on chromosomal data, and in recent years, with the involvement of data from molecular phylogenetics [17,18,20,21].

Although this group has attracted the attention of numerous researchers [4,17,18,19,20,21,22,23,24,25,26,27,28], a large number of unresolved taxonomic problems persist in *Agrodiaetus*. One of these problems is the taxonomic structure of the subgenus as a whole, namely the division of the subgenus into natural monophyletic lineages [22].

This subgenus has been studied relatively well with respect to molecular markers, and for many species, multi-locus molecular data are available, including such genes as mitochondrial *COI*, *tRNA-leu*, *COII*, *cytochrome b* and *NADH dehydrogenase* sequences and nuclear *5.8S rDNA*, *ITS2*, *28S rDNA* and *EF1-α* sequences [4,17,18,27,29,30,31]. At the same time, for many taxa, especially for rare species from Turkey, Iran, Pakistan and Afghanistan, only mitochondrial DNA barcodes are available [32], or the molecular data are absent.

In this work, we

(1) obtain and analyze standard mitochondrial DNA barcodes for five deviated and most enigmatic taxa of the subgenus *Agrodiaetus*: *P. muellerae* Eckweiler, 1997 from Pakistan, *P. afghanicus* (Forster, 1973) and *P. frauvartianae* Balint, 1997 from Afghanistan, *P. bogra* Evans, 1932 from Afghanistan and Iran, and *P. anticarmon* (Koçak, 1983) (= *charmeuxi* Pages, 1984) from SE Turkey;

(2) analyze the partitioning of the subgenus *Agrodiaetus* into species groups by using three data sets. The first data set is represented by short mitochondrial DNA barcodes for all analyzed samples. The second and third data sets are represented by a combination of short mitochondrial DNA barcodes for part of the taxa with longer mitochondrial sequences *COI* + *tRNA-Leu* + *COII* (data set 2) and with longer mitochondrial *COI* + *tRNA-Leu* + *COII* and nuclear *5.8S rDNA* + *ITS2* + *28S rDNA* sequences (data set 3) for the remaining species;

(3) show that the DNA barcoding approach (data set 1) failed to reveal the phylogenetic structure of the subgenus, whereas a combined analysis of the mitochondrial and nuclear sequences (data sets 2 and 3) revealed the species groups and the position within these species groups, even for taxa for which only mitochondrial sequences were available;

(4) provide a list of the species groups of the subgenus *Agrodiaetus*; and

(5) discuss the status and taxonomic position of *P. muellerae*, *P. afghanicus*, *P. frauvartianae*, *P. bogra* and *P. anticarmon*.

## 2. Materials and Methods

Standard mitochondrial DNA barcodes (658 bp fragments of the *cytochrome c oxidase subunit I* gene) were obtained for five samples of *P. afghanicus*, one sample of *P. anticarmon* (= *charmeuxi*), six samples of *P. bogra*, seven samples of *P. frauvartianae* and one sample of *P. muellerae* (Table 1). The specimens (except for samples BPAL2125–BPAL2128) were processed at the Department of Karyosystematics of the Zoological Institute of the Russian Academy of Sciences. DNA extraction from a single leg removed from each specimen was performed using the QIAamp DNA Investigator Kit (Qiagen, Venlo, The Netherlands) according to the manufacturer’s protocol. Since standard lepidopteran barcode primers [7] failed to amplify a sufficient product, two self-designed forward primers (Nz_COI_b—TAC AAT TTA TCG CTT ATA AACTCA; DRD4F—TAGAAAATGGAGCAGGAA) and two reverse primers (MH-MR1 [33] and Nancy [34]) were used for DNA amplification and resulted in a 671 bp fragment of the mitochondrial cytochrome oxidase I gene (*COI*). The PCR amplifications were performed in a 50 µL reaction volume containing ca. 10–20 ng genomic DNA and 0.5 mM each of forward and reverse primer, 1 mM dNTPs, 10× PCR Buffer (0.01 mM Tris-HCl, 0.05 M KCl, 0.1% Triton X-100: pH 9.0), 1 unit Taq DNA Polymerase (Thermo Fisher Scientifics, Waltham, MA, USA), 5 mM MgCl_2_. The temperature profile was as follows: initial denaturation at 94 °C for 1 min, followed by 30 cycles of denaturation at 94 °C for 45 s, annealing at 50 °C for 45 s and extension at 72 °C for 1 min, with a final extension at 72 °C for 10 min. Amplified fragments were purified using GeneJET Gel Extraction Kit (Thermo Fisher Scientifics, Waltham, MA, USA). Purification was carried out according to the manufacturer’s protocol. The success of PCR amplification and purification was evaluated through electrophoresis of the products in 1% agarose gel. The purified PCR product was used for direct sequencing. Sequencing of the double stranded product was carried out at the Research Resource Center for Molecular and Cell Technologies (St. Petersburg State University).

Samples BPAL2125–BPAL2128 were processed at the Canadian Centre for DNA Barcoding (CCDB, Biodiversity Institute of Ontario, University of Guelph), using their standard high-throughput protocol described by deWaard et al. [35].

A comparison of the obtained *COI* barcodes revealed 11 unique haplotypes within the five species studied (Table 1).

For the other 130 species and well-differentiated subspecies of the subgenus *Agrodiaetus*, all available sequences of the mitochondrial (*COI*, *leu-tRNA* complete and *COII* partial) and nuclear (*5.8S rDNA* partial gene, *ITS2* complete and *28S rDNA* partial) loci were extracted from GenBank (see Supplementary Material S1 for the GenBank numbers). The sequences of each locus (gene) were aligned separately by using the ClustalW algorithm, and then, the alignments were checked and corrected manually using BioEdit [36]. Since within *Agrodiaetus*, the previous phylogenetic analyses of the nuclear sequences *5.8S rDNA* + *ITS2* + *28S rDNA* recovered clades, which were mostly congruent with those obtained from analyses of the mitochondrial genes *COI* + *COII* [25], the nuclear and mitochondrial sequences were concatenated for subsequent phylogenetic study. This concatenation was then combined with the 11 unique haplotypes revealed within the five species studied (Table 1). The representatives of the closely related subgenus *Polyommatus* (*P. icarus* Rottemburg, 1775, *P. erotides* Staudinger, 1892 and *P. hunza* Grum-Grshimailo, 1890) [4] were also included in the analysis. To root the tree, we used representatives of a more distant genus *Lysandra* (*L. bellargus*, *L. punctifera* and *L. coridon*) [4]. The final matrix consisted of 147 taxa, of which 141 taxa represented our target species, and 6 taxa represented the outgroup. For 87 of these 147 taxa, the matrix contained data for both mitochondrial and nuclear genes. For 60 of these 147 taxa, only mitochondrial gene(s) were available.

Since the *ITS2* sequence has multiple indels, which are highly specific on the species level, it provides additional information for phylogenetic analysis; therefore, we treated all *ITS2* indels as binary characteristics (insertion—1, deletion—0). The final concatenated alignment had a length of 2948 nucleotides (*COI*—1–1539 bp, *leu-tRNA*—1540–1604 bp, *COII*—1605–2281 bp, *5.8S rDNA* + *ITS2* + *28S rDNA*—2282–2948 bp) and 23 binary characteristics.

**Figure 1:**
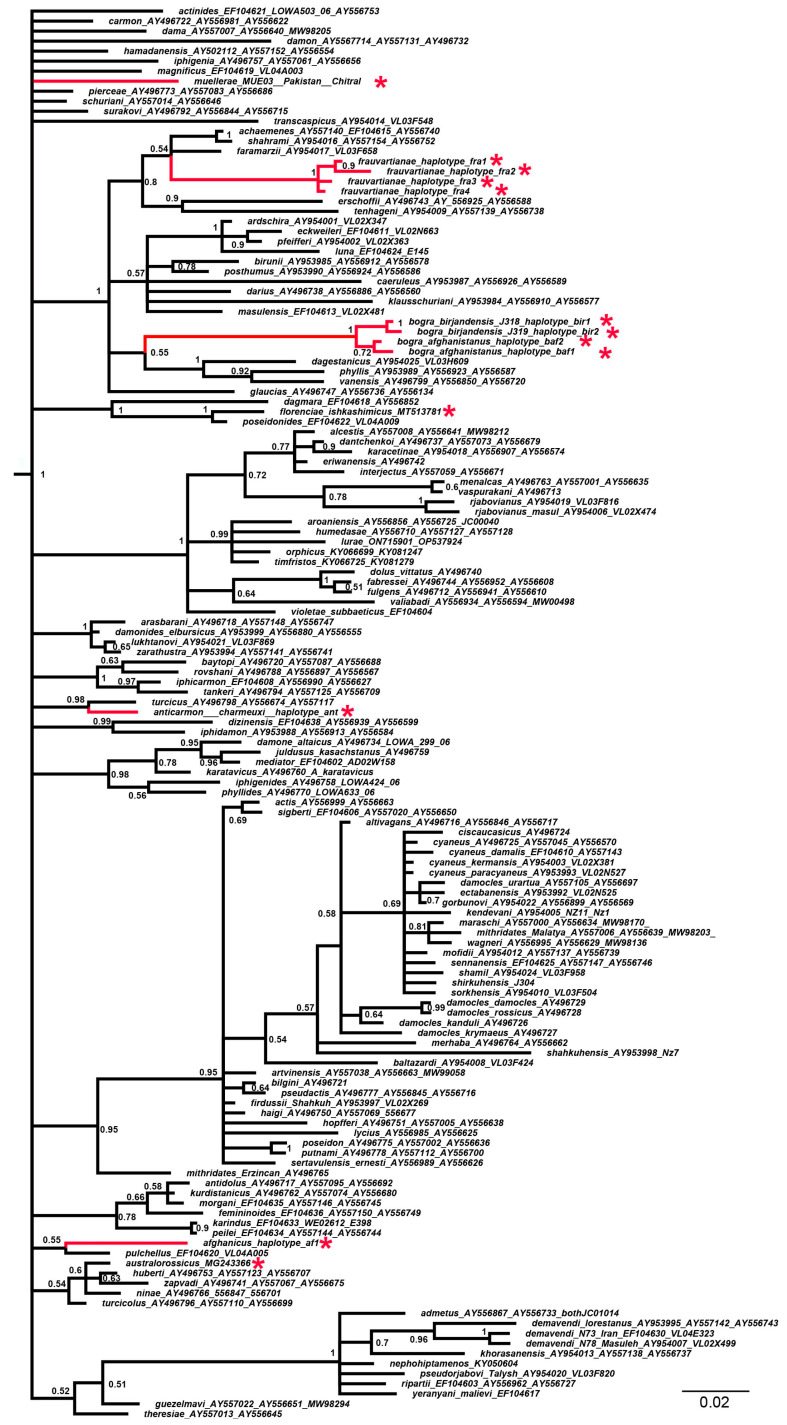
The Bayesian tree of *Agrodiaetus* species based on analysis of the short mitochondrial *COI* barcodes. Numbers at nodes indicate Bayesian posterior probabilities (BPP) (higher than 0.5). Red asterisks indicate samples for which longer mitochondrial and/or nuclear sequences were unavailable. The subgenus *Polyommatus* sensu stricto was found to be a statistically supported (BPP = 1) clade, sister to *Agrodiaetus* (not shown). The genus *Lysandra* (not shown) was used to root the tree.

**Figure 2:**
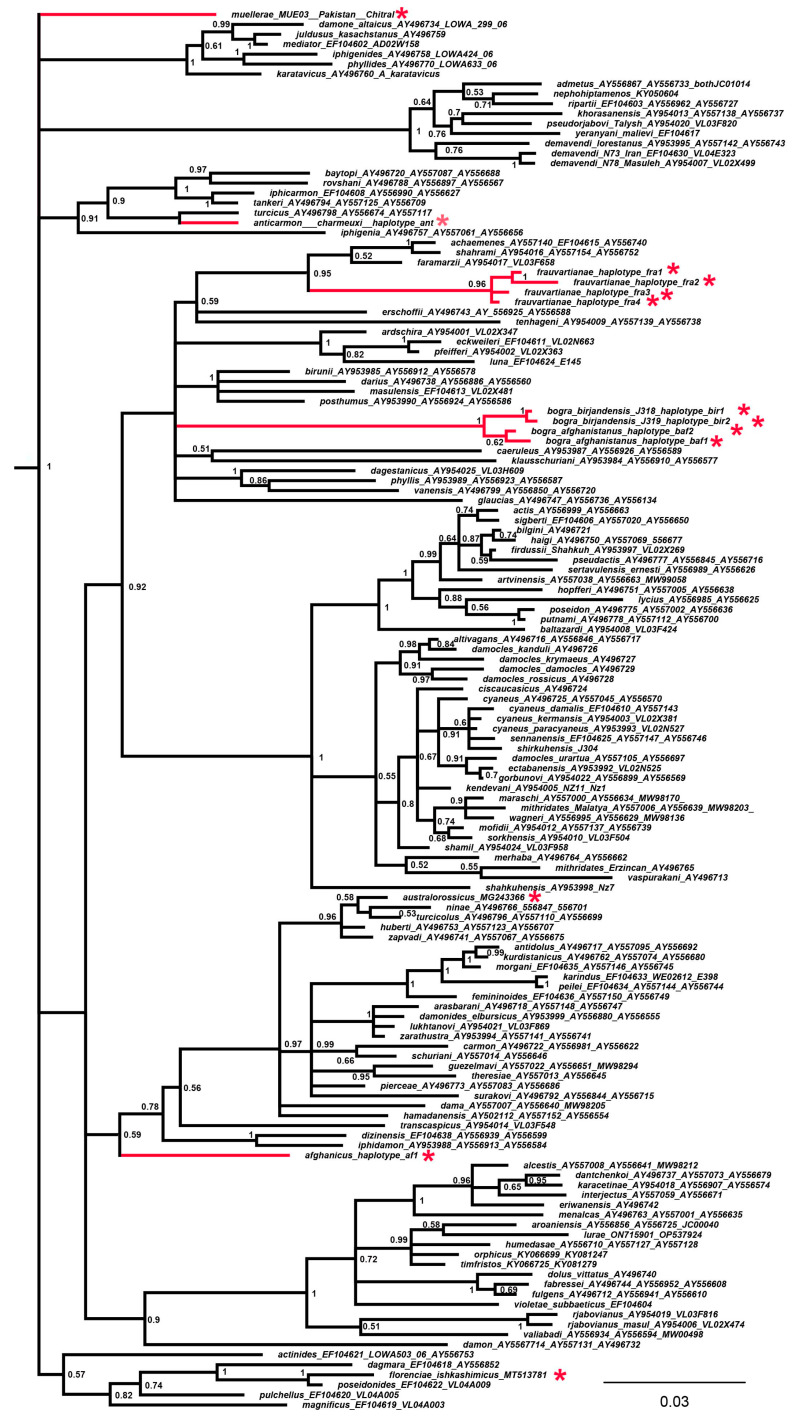
The Bayesian tree of *Agrodiaetus* species based on combined analysis of the mitochondrial *COI* + *tRNA-Leu* + *COII* sequences. Numbers at nodes indicate Bayesian posterior probability higher than 0.5. Red asterisks indicate species for which only short DNA barcodes were available. The subgenus *Polyommatus* sensu stricto was found to be a statistically supported (BPP = 1) clade, sister to *Agrodiaetus* (not shown). The genus *Lysandra* (not shown) was used to root the tree.

**Figure 3:**
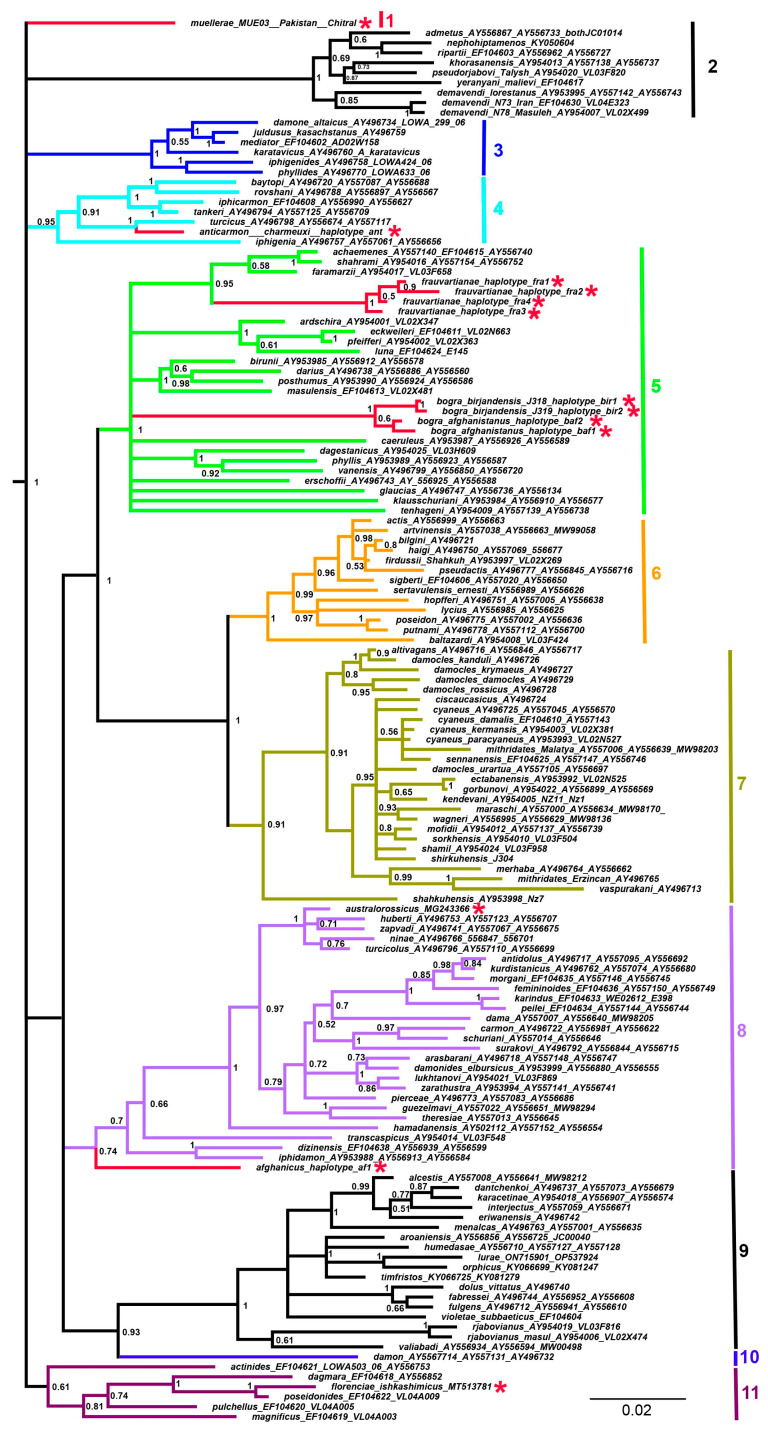
The Bayesian tree of *Agrodiaetus* species based on combined analysis of the mitochondrial *COI* + *tRNA-Leu* + *COII* and nuclear *5.8S rDNA* + *ITS2* + *28S rDNA* sequences. Numbers at nodes indicate Bayesian posterior probabilities higher than 0.5. Red asterisks indicate species for which only short DNA barcodes were available. The species groups are denoted as 1–11. The subgenus *Polyommatus* sensu stricto was found to be a statistically supported (BPP = 1) clade, sister to *Agrodiaetus* (not shown). The genus *Lysandra* (not shown) was used to root the tree.

Three data sets were prepared from the final concatenated alignment. In data set 1, for all 147 samples studied, only short *COI* barcodes were presented. In data set 2, for 13 samples (shown with a red asterisk (*) in Figure 1, Figure 2 and Figure 3), only short *COI* barcodes were available, and for the remaining 134 samples, the longer mitochondrial sequence *COI* + *tRNA-Leu* + *COII* was presented. In data set 3, the mitochondrial matrix (data set 2) was supplemented with *5.8S rDNA* + *ITS2* + *28S rDNA* nuclear sequences for 87 samples.

Substitution models were inferred separately for each gene (locus) using jModeltest, version 2 [37]. Bayesian analysis was conducted using MrBayes 3.2 [38] on four molecular (*COI*, *COII*, *leu-tRNA* and *5.8S rDNA* + *ITS2* + *28S rDNA* genes) and one “standard” (binary) partitions using 20,000,000 generations. The command blocks for the first, second and third data sets are presented in Supplementary Material S2.

## 3. Results

The analysis of the DNA barcodes (Figure 1) did not reveal the structure of the subgenus *Agrodiaetus*. Only 33 statistically supported clades (posterior probability >= 0.9) were recovered, and the position of the majority of species—particularly of our target taxa *P. muellerae*, *P. afghanicus*, *P. frauvartianae*, *P. bogra* and *P. anticarmon*—was unresolved. This was manifested in the facts that (1) the relationships with sister species were not identified (*P. muellerae*); (2) the sister relationships had low support (*P. afghanicus*, *P. frauvartianae*, *P. bogra*); and (3) it was not clear which species groups the target species belonged to (*P. muellerae* and *P. afghanicus*).

The combined analysis of mitochondrial (Figure 2) and mitochondrial + nuclear sequences (Figure 3) resulted in a significant increase in the resolution of the phylogenetic tree, with 53 highly supported clades (posterior probability >= 0.9) for data set 2 and with 65 highly supported clades (posterior probability >= 0.9) for data set 3. Thus, adding additional mitochondrial and nuclear loci resulted in an increased number of highly supported clades, which was not unexpected. A more interesting observation is that this approach resulted in increased support and tree position detection for those branches for which additional mitochondrial and nuclear data were not available (shown with red asterisks on the trees).

It was established that the taxon *P. frauvartianae* was included in the same clade together with the species *P. faramarzii* Skala 2001, *P. shahrami* Skala, 2002 and *P. achaemenes* Skala, 2002 (Figure 3), although this relationship had extremely low support (0.54) on the DNA barcode tree (Figure 1). It was shown that *P. anticarmon* was not only a sister species to *P. turcicus* (Koçak, 1977) (Figure 1) but that both of these taxa were members of the same clade together with *P. iphigenia* (Herrich-Schäffer, 1847) (Figure 3). The position of the taxon *P. australorossicus* Lukhtanov and Dantchenko, 2017 on the DNA barcode tree (Figure 1) was unclear due to low support. On the combined tree (Figure 2 and Figure 3), this species was placed with high support in the same clade along with *P. hamadanensis* (de Lesse, 1959).

**Figure 4:**
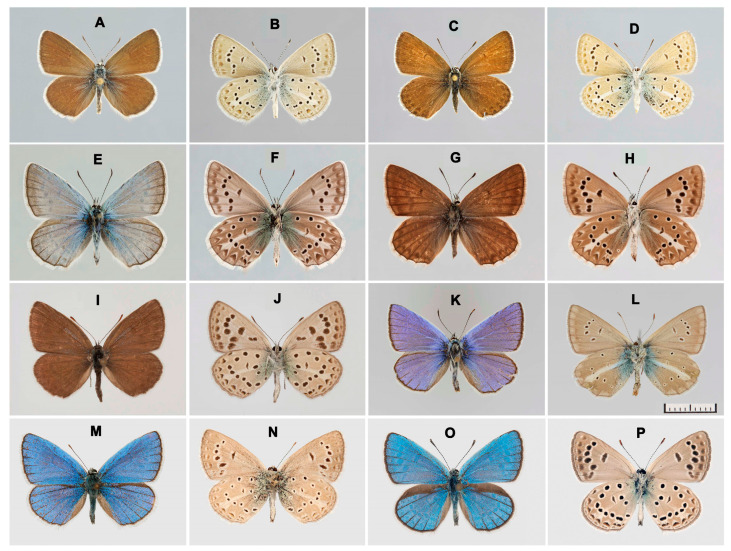
The species studied. (**A**–**D**). *P*. (*A*.) *afghanicus*, Afghanistan, Koh-i-Baba, Band-i-Amir, 34.8294° N, 67.1805° E, 2900–3000 m., 2 July 2009, Yu. Skrylnik leg. (**A**,**B** male; **C**,**D** female). (**E**–**H**). *P*. (*A*.) *muellerae*, Pakistan, Chitral, Birmogh Lasht, 35.8981° N, 71.7712° E, 2600–3000 m, 1 July 2001, leg. V. Tuzov (**E**,**F** male, mue02; **G**,**H** female, mue03). (**I**,**J**). *P*. (*A*.) *frauvartianae*, male, Afghanistan, Kotale Altimur, 2900 m, 10 July 1973, leg. Dr. Resholt. (**K**,**L**). *P*. (*A*.) *anticarmon* (= *charmeuxi*), male CHAR01, Turkey, Hakkari Prov., vic. Üzümcū 1300 m, 28 June–4 July 1976. (**M**,**N**). *P*. (*A*.) *bogra afghanistanus*, Afghanistan, Prov. Bamiyan, vic. Panjao, 2200 m, 26–28 June 1970, leg. C. Naumann. (**O**,**P**). *P*. (*A*.) *bogra birjandensis*, Iran, Khorasan, Birjand, Mazarkahi, 2100–2200 m, 16–17 May 2000, leg. W. Eckweiler.

Our analysis revealed 11 major lineages, shown with different colors (Figure 3). Two lineages were represented by a single species. Seven lineages were highly supported (posterior probability value from 0.91 to 1). The target species *P. afghanicus* (Figure 4A–D) appeared as a lineage distantly related to the lineage *P. antidolus*–*P. iphidamon* (species group 8); however, the sister relationship between them was weakly supported. *Polyommatus muellerae* (Figure 4E–H) appeared as a distinct lineage (species group 1). The target species *P. frauvartianae* (Figure 4I,J 12), *P. bogra* (Figure 4M–P) and *P. anticarmon* (Figure 4K–L) appeared as members of species groups 5 and 4.

## 4. Discussion

The methodology proposed by Talavera et al. [8] allowed us to identify the positions on the phylogenetic tree for the rare south Central Asian taxa, for which the molecular data were available only in the form of short DNA barcodes. The data obtained represent an empirical test of the previously proposed methodology [8] and provide the opportunity to discuss in more detail the taxonomy of the species studied.

*Polyommatus frauvartianae* from Afghanistan was described as a distinct species by Balint [39], but then, on the basis of external morphological similarity, it was interpreted as a subspecies of the Iranian-Turkmen species *P. glaucias* (Lederer, 1871) [19]. Our data unequivocally show that this is an independent species, phylogenetically distant from *P. glaucias*, but undoubtedly having a relationship with *P. faramarzii*, *P. shahrami* and *P. achaemenes*, endemics of the Zagros Mts in Iran. Of the last three species, *P. frauvartianae* is well distinguished by the brown (not blue) coloration of the upper side of the wings in males. Thus, these data shed light on the origin of the enigmatic Iranian taxa *P. faramarzii*, *P. shahrami* and *P. achaemenes*, which, having dot-like distribution ranges in the Zagros, did not show close relationships with any other species of the subgenus *Agrodiaetus*. The new data show that the four species *P. faramarzii*, *P. shahrami*, *P. achaemenes* and *P. frauvartianae* are members of the same phylogenetic sub-lineage, spread over a wide area from western Iran to central Afghanistan.

Our data show the conspecificity of two geographically distant population groups identified as *P. bogra birjandensis* Eckweiler, 2003 (E Iran) and *P. bogra afghanistanus* (Forster, 1972) (Afghanistan) [19]. Thus, the polytypic concept of the *P. bogra* species, as proposed by Eckweiler and Bozano [19], is confirmed.

The taxon *P. anticarmon* is also a subject of taxonomic debates [19]. Butterflies of this taxon inhabiting SE Turkey are similar in appearance to *P. turcicus* from NE Turkey and Armenia, differing in larger size. In addition, there is a difference in ecological preferences between *P. turcicus* and *P. anticarmon*: while *P. turcicus* is an alpine species, *P. anticarmon* is found at relatively low altitudes. Our data show that *P. anticarmon* is indeed closely related to *P. turcicus*. According to Eckweiler and Bozano [19], the taxon *P. charmeuxi* described from SE Turkey is a synonym of *P. anticarmon*.

In addition, the phylogenetic analysis conducted allows us to discuss another very controversial issue of *Agrodiaetus* taxonomy, namely the division of the subgenus into groups of species. In most cases, species delimitation and recognition of monophyletic species groups within *Agrodiaetus* are difficult because of the low number of differentiated morphological characteristics. The morphology of male genitalia is uniform throughout most of the species, with a few exceptions [19,40,41]. Some *Agrodiaetus* species show considerable variability in male wing color, both in visible and ultraviolet wavelength ranges [29]. Despite this variation, it is difficult to use this characteristic for phylogenetic purposes, since in the great majority of species, it is represented by a plesiomorphic state (blue color), and the derived states are found mostly as unique apomorphies characterizing single species but not species groups [29].

The same can be said of chromosomal characteristics. Although the chromosome numbers in *Polyommatus* (*Agrodiaetus*) possess a strong phylogenetic signal [18], generally, the karyotypes are extremely variable on an inter-specific level, and they are found mostly as unique apomorphies characterizing single species but not species groups [18,20,21,30,42,43,44,45].

de Lesse [20,21] divided *Agrodiaetus* into three species complexes based on male coloration and the presence/absence of well-developed tufts formed by androconial scales. In the classification by Hesselbarth et al. [46], *Agrodiaetus* was divided into eight species groups (*actis*, *admetus*, *carmon*, *damon*, *damone*, *dolus*, *poseidon* and *transcaspicus*) named after their oldest members. Eckweiler and Häuser [22] recognized the *admetus* and *dolus* groups but argued that the available evidence was too weak to support the remaining groups. Instead, they erected a more inclusive *damon* group, which combined the membership of Hesselbarth et al.’s [46] *actis*, *carmon*, *damon*, *damone* and *transcaspicus* groups with some species from the *poseidon* group. The remainder of the *poseidon* group was renamed as the *dama* group, and three additional species groups—the *dagmara*, *erschoffii* (= *Paragrodiaetus* Rose and Schurian, 1977) and *iphigenides* groups—were erected to accommodate species from eastern Iran and central Asia, which had not been considered by Hesselbarth et al. [46].

Similarly, Balint [39] proposed a more fractional division and separated *Agrodiaetus* into the following groups: *actinides*, *actis*, *admetus*, *carmon*, *dama*, *damon*, *damone*, *dolus*, *nadira*, *poseidon*, *poseidonides* and *transcaspicus*. He also considered *Paragrodiaetus* as a complex separated from *Agrodiaetus* and divided *Paragrodiaetus* into two groups: *erschoffii* and *glaucias*.

Molecular studies [17,18,27,29,30] revealed that the previously recognized species groups [20,21,39,46] were mostly non-monophyletic assemblages. This resulted in the creation of a new division of the subgenus *Agrodiaetus*, comprising 10 clades: *damocles*, *actis*, *erschoffii*, *carmon*, *admetus*, *dolus*, *damone*, *magnificus*, *iphigenia* and *damon* [18].

Here, using the analysis of additional taxa and additional molecular markers, we demonstrate that the subgenus *Agrodiaetus* consists of 11 lineages and 135 species (Appendix A). In particular, we also show that the enigmatic taxon *P*. (*A*.) *muellerae* from Pakistan represents a distinct evolutionary lineage and cannot be included in the previously recognized species groups.

Koçak and Kemal [47] divided *Agrodiaetus* into 13 subsections and proposed the following names for these subsections: *Actisia*, *Admetusia*, *Antidolus*, *Dagmara*, *Damaia*, *Juldus*, *Musa*, *Paragrodiaetus*, *Peileia*, *Phyllisia*, *Transcaspius*, *Xerxesia* and *Agrodiaetus* s.str. Here, we demonstrate that these subsections do not reflect the evolutionary and taxonomic structure revealed using molecular markers (see the list below). Three species groups discovered in our study (*erschoffii*, *damocles* and *carmon*) are represented by two (*erschoffii*, *damocles* groups) and even by four (*carmon* group) names from the list proposed by Koçak and Kemal [47], whereas five species groups have no names, and only five names are unambiguously associated with the species groups (one name corresponds to one species group). According to the Code of Zoological Nomenclature (ICZN 10.4) [48], “a uninominal name proposed for a genus-group division of a genus, even if proposed for a secondary (or further) subdivision, is deemed to be a subgeneric name even if the division is denoted by a term such as ‘section’ or ‘division’”. Thus, the names proposed by Koçak and Kemal [47] should be considered subgeneric names and therefore subjective junior synonyms of *Agrodiaetus*, since the subgeneric rank of *Agrodiaetus* is well founded [4].

Finally, we should note that although the approach used [8] markedly improved the resolution of the phylogenetic tree, some clades—in particular the *carmon* and *magnificus* species groups—have low support. Obviously, further studies based on the analysis of more genes are needed in order to obtain a fully resolved phylogeny of the subgenus *Agrodiaetus*.

## 5. Conclusions

1. We show that the DNA barcoding approach failed to reveal the phylogenetic structure of the subgenus *Agrodiaetus*, whereas a combined analysis of the mitochondrial and nuclear sequences revealed the species groups and the position within these species groups, even for taxa for which only short DNA barcodes were available.

2. The Afghani taxon *Polyommatus frauvartianae* is a distinct species, most closely related to west Iranian endemics *P. faramarzii*, *P. shahrami* and *P. achaemenes*.

3. *P. bogra birjandensis* (E Iran) and *P. bogra afghanistanus* (Afghanistan) are confirmed as members of the polytypic species *P. bogra*.

4. *Polyommatus anticarmon* (= *charmeuxi*) is identified as a taxon, which is a sister to *P. turcicus*.

5. The enigmatic Pakistani taxon *P. muellerae* represents a distinct evolutionary lineage and cannot be included in previously recognized species groups.

6. The subgenus *Agrodiaetus* consists of 11 lineages (species groups).

7. We confirm the previous conclusion [22] that the following subgeneric names are subjective junior synonyms of *Agrodiaetus*:

*Actisia* Koçak and Kemal, 2001;

*Admetusia* Koçak and Seven, 1998;

*Antidolus* Koçak and Kemal, 2001;

*Dagmara* Koçak and Kemal, 2001;

*Damaia* Koçak and Kemal, 2001;

*Hirsutina* Tutt, 1909;

*Juldus* Koçak and Kemal, 2001;

*Musa* Koçak and Kemal, 2001;

*Paragrodiaetus* Rose and Schurian, 1977;

*Peileia* Koçak and Kemal, 2001;

*Phyllisia* Koçak and Kemal, 2001;

*Transcaspius* Koçak and Kemal, 2001;

*Xerxesia* Koçak and Kemal, 2001.

## Figures and Tables

**Table 1:** List of the studied samples and obtained *COI* sequences.

Species	Laboratory ID	GeneBank	Haplotype	Country	Locality
*P.* (*A.*) *afghanicus*	AF05	OR413713	*af1*	Afghanistan	Koh-i-Baba, Band-i-Amir, 34.8294° N, 67.1805° E, 2900–3000 m, 2 July 2009, Yu. Skrylnik leg.
	BPAL2125	OR413714	*af1*	Afghanistan	near Kabul, July 2010, I. Pljushch leg.
	BPAL2126	OR413715	*af1*	Afghanistan	the same locality
	BPAL2127	OR413716	*af1*	Afghanistan	the same locality
	BPAL2128	OR413717	*af1*	Afghanistan	the same locality
*P.* (*A.*) *anticarmon* (= *charmeuxi*)	CHAR01	OR424389	*ant*	Turkey	Hakkari Prov., vic. Üzümcū 1300 m, 28 June – 4 July 1976
*P.* (*A.*) *bogra afghanistanus*	XX21	OR424390	*baf1*	Afghanistan	Bamyan prov., 8 km S Bamyan, 2700 m, 31 May 2010, O. Pak leg.
	AAF02	OR424391	*baf2*	Afghanistan	the same locality
	AAF10	OR424392	*baf1*	Afghanistan	Bamiyan prov., 34.2155° N, 66.8994° E, 2545 m, 23 June 2016, O. Pak leg.
	AAF11	OR424393	*baf1*	Afghanistan	the same locality
*P.* (*A.*) *bogra birjandensis*	J318	OR413718	*bir1*	Iran	South Khorasan Prov., 26 km N of Birjand, 1900–2000 m, 14 July 2007, V. Lukhtanov leg.
	J319	OR413719	*bir2*	Iran	the same locality
*P.* (*A.*) *frauvartianae*	AAF01	OR424394	*fra1*	Afghanistan	Bamiyan prov., Yakawlang District, Bandi-Amir env., 3300 m, 3 August 2011, O. Pak leg.
	AAF03	OR424395	*fra2*	Afghanistan	the same locality
	AAF04	OR424396	*fra1*	Afghanistan	the same locality
	AAF05	OR424397	*fra1*	Afghanistan	the same locality
	AAF07	OR424398	*fra3*	Afghanistan	the same locality
	AAF08	OR424399	*fra4*	Afghanistan	Bamiyan prov., Panjab District, Kohi-Baba Mts., Rashak Mts., Shatu Pass, 3490 m, 6 August 2011, O. Pak leg.
	AAF09	OR424400	*fra1*	Afghanistan	the same locality
*P.* (*A.*) *muellerae*	MUE03	OR413720	*mu1*	Pakistan	Chitral, Birmogh Lasht, 35.8981° N, 71.7712° E, 2600–3000 m, 1 July 2001, leg. V. Tuzov

## Data Availability

All the analyzed DNA sequences are available via the GenBank links provided.

## Supplementary Materials

The following supporting information can be downloaded at: https://www.mdpi.com/article/10.3390/insects14090769/s1, Supplementary Material S1. The final alignment of the analyzed samples with their GenBank numbers (file in the FASTA format). Supplementary Material S2. The command blocks used for the Bayesian analysis of the first, second and third data sets.

## Appendix A

The checklist of the species groups and species of *Agrodiaetus* is provided below. The type localities and brief information on the ranges of the species are provided in the work of Eckweiler and Bozano [19].

**Checklist of the species groups and species of *Agrodiaetus***

(1) ***muellerae* Eckweiler, 1997 species group**

*muellerae* Eckweiler, 1997

(2) ***admetus* (Esper, 1783) species group**

*admetus* (Esper, 1783) Type species of *Admetusia* Koçak and Seven, 1998

*nephohiptamenos* (Brown and Coutsis, 1978)

*ripartii* (Freyer, 1830)

*khorasanensis* (Carbonell, 2001)

*pseudorjabovi* Lukhtanov, Dantchenko, Vishnevskaya and Saifitdinova, 2015

*yeranyani* (Dantchenko and Lukhtanov, 2005)

*demavendi* (Pfeiffer, 1938)

(3) ***damone* (Eversmann, 1841) species group**

*damone* (Eversmann, 1841)

*juldusus* (Staudinger, 1886) Type species of *Juldus* Koçak and Kemal, 2001

*mediator* Dantchenko and Churkin, 2003

*karatavicus* Lukhtanov, 1990

*iphigenides* (Staudinger, 1886)

*phyllides* (Staudinger, 1886)

(4) ***iphigenia* (Herrich-Schäffer, 1847) species group**

*baytopi* (de Lesse, 1959)

*rovshani* Dantchenko and Lukhtanov, 1994

*iphicarmon* Eckweiler and Rose, 1993

*tankeri* (de Lesse, 1960)

*turcicus* (Koçak, 1977)

*anticarmon* (Koçak, 1983) (= *charmeuxi* Pages, 1984)

*iphigenia* (Herrich-Schäffer, 1847)

(5) ***erschoffii* (Lederer, 1869) species group**

*achaemenes* Skala, 2002

*shahrami* Skala, 2001

*faramarzii* Skala, 2001

*frauvartianae* Balint, 1997

*ardschira* (Brandt, 1938)

*eckweileri* ten Hagen, 1998

*pfeifferi* (Brandt, 1938)

*luna* Eckweiler, 2002

*birunii* Eckweiler and ten Hagen, 1998

*darius* Eckweiler and ten Hagen, 1998

*posthumus* (Christoph, 1877)

*masulensis* ten Hagen and Schurian, 2000

*bogra* Evans, 1932

*caeruleus* (Staudinger, 1871)

*dagestanicus* (Forster, 1960)

*phyllis* (Christoph, 1877) Type species of *Phyllisia* Koçak and Kemal, 2001

*vanensis* (de Lesse, 1957)

*erschoffii* (Lederer, 1869)

*glaucias* (Lederer, 1871) Type species of *Paragrodiaetus* Rose and Schurian, 1977

*klausschuriani* ten Hagen, 1999

*tenhageni* Schurian and Eckweiler, 1999

(6) ***actis* (Herrich-Schäffer, 1851) species group**

*actis* (Herrich-Schäffer, 1851) Type species of *Actisia* Koçak and Kemal, 2001

*artvinensis* (Carbonell, 1997)

*bilgini* (Dantchenko and Lukhtanov, 2002)

*haigi* (Dantchenko and Lukhtanov, 2002)

*firdussii* (Forster, 1956)

*pseudactis* (Forster, 1960)

*sigberti* (Olivier et al. 2000) (? *athis* Freyer, 1851)

*sertavulensis* (Koçak, 1979)

*hopfferi* (Herrich-Schäffer, 1851)

*lycius* (Carbonell, 1996)

*poseidon* (Herrich-Schäffer, 1851)

*putnami* (Dantchenko and Lukhtanov, 2002)

*baltazardi* (de Lesse, 1962)

(7) ***damocles* (Herrich-Schäffer, 1844) species group**

*altivagans* (Forster, 1956)

*damocles* (Herrich-Schäffer, 1844)

*ciscaucasicus* (Forster, 1956)

*cyaneus* (Staudinger, 1899)

*cyaneus musa* Koçak and Hosseinpour, 1996 Type species of *Musa* Koçak and Kemal, 2001

*cyaneus xerxes* (Staudinger, 1899) Type species of *Xerxesia* Koçak and Kemal, 2001

*sennanensis* (de Lesse, 1959)

*urartua* (Carbonell, 2003)

*ectabanensis* (de Lesse, 1964)

*gorbunovi* (Dantchenko and Lukhtanov, 1994)

*kendevani* (Forster, 1956)

*maraschi* (Forster, 1956)

*wagneri* (Forster, 1956)

*mofidii* (de Lesse, 1963)

*sorkhensis* Eckweiler, 2003

*shamil* (Dantchenko, 2000)

*shirkuhensis* ten Hagen and Eckweiler, 2001

*merhaba* (de Prins, van der Poorten, Borie, van Oorschot, Riemis and Coenen 1991)

*mithridates* (Staudinger, 1878)

*vaspurakani* (Lukhtanov and Dantchenko, 2003)

*shahkuhensis* (Lukhtanov, Shapoval and Dantchenko, 2008)

*barmifiruze* (Carbonell, 2000) (no molecular data)

*cilicius* (Carbonell, 1998) (no molecular data)

*sephidarensis* Karbalaye, 2008 (no molecular data)

*esfahensis* (Carbonell, 2000) (no molecular data)

(8) ***carmon* (Herrich-Schäffer, 1851) species group**

*australorossicus* Lukhtanov and Dantchenko, 2017

*huberti* (Carbonell, 1993)

*zapvadi* (Carbonell, 1993)

*ninae* (Forster, 1956)

*turcicolus* (Koçak, 1977)

*antidolus* (Rebel, 1901) Type species of *Antidolus* Koçak and Kemal, 2001

*kurdistanicus* (Forster, 1961)

*morgani* (Le Cerf, 1909)

*femininoides* (Eckweiler, 1987)

*karindus* (Riley, 1921)

*peilei* Bethune-Baker, 1921 Type species of *Peileia* Koçak and Kemal, 2001

*dama* (Staudinger, 1892) Type species of *Damaia* Koçak and Kemal, 2001

*carmon* (Herrich-Schäffer, 1851)

*schuriani* (Rose, 1978)

*surakovi* Dantchenko and Lukhtanov, 1994

*arasbarani* (Carbonell and Naderi, 2000)

*damonides* (Staudinger, 1899) (= *elbursicus* Forster, 1956)

*lukhtanovi* (Dantchenko, 2004)

*zarathustra* Eckweiler, 1997

*pierceae* (Lukhtanov and Dantchenko, 2002)

*guezelmavi* Olivier, Puplesiene, van der Poorten, de Prins and Wiemers, 1999

*theresiae* Schurian, van Oorschot and van den Brink, 1992

*hamadanensis* (de Lesse, 1959)

*transcaspicus* (Heyne, 1895) Type species of *Transcaspius* Koçak and Kemal, 2001

*dizinensis* (Schurian, 1982)

*iphidamon* (Staudinger, 1899)

*afghanicus* (Forster, 1973)

*larseni* (Carbonell, 1994) (no molecular data)

*zardensis* Schurian and ten Hagen, 2001 (no molecular data)

*alibali* (Carbonell, 2015) (no molecular data)

*kashani* Eckweiler, 2013 (no molecular data)

*lori* Eckweiler, 2013 (no molecular data)

(9) ***dolus* (Hübner, 1823) species group**

*alcestis* (Zerny, 1932)

*dantchenkoi* Lukhtanov and Wiemers, 2003

*karacetinae* (Lukhtanov and Dantchenko, 2002)

*interjectus* (de Lesse, 1960)

*eriwanensis* (Forster, 1960)

*menalcas* (Freyer, 1837)

*aroaniensis* (Brown, 1976)

*humedasae* (Toso and Balletto, 1976)

*lurae* Parmentier, Vila and Lukhtanov, 2022

*orphicus* Kolev, 2005

*timfristos* Lukhtanov, Vishnevskaya and Shapoval, 2016

*dolus* (Hübner, 1823)

*fabressei* (Oberthür, 1910)

*fulgens* (de Sagarra,1925)

*violetae* (Gómez-Bustillo, Expósito Hermosa and Martínez Borrego, 1979)

*rjabovianus* (Koçak, 1980)

*valiabadi* (Rose and Schurian, 1977)

(10) ***damon* ([Denis and Schiffermüller], 1775) species group**

*damon* (Denis and Schiffermüller, 1775) Type species of *Agrodiaetus*, type species of *Hirtusina* Tutt, 1909

(11) ***magnificus* (Grum-Grshimailo, 1885) species group**

*actinides* (Staudinger, 1886)

*dagmara* (Grum-Grshimailo, 1888) Type species of *Dagmara* Koçak and Kemal, 2001

*florenciae* (Tytler, 1926)

*poseidonides* (Staudinger, 1886)

*pulchellus* (Bernardi, 1951)

*magnificus* (Grum-Grshimailo, 1885)

## References

[1] Rubinoff D., Holland B.S. (2005). Between two extremes: Mitochondrial DNA is neither the panacea nor the nemesis of phylogenetic and taxonomic inference. Syst. Biol..

[2] Will K.W., Mishler B.D., Wheeler Q.D. (2005). The perils of DNA barcoding and the need for integrative taxonomy. Syst. Biol..

[3] Knowles L.L., Carstens B.C. (2007). Delimiting species without monophyletic gene trees. Syst. Biol..

[4] Talavera G., Lukhtanov V.A., Pierce N.E., Vila R. (2013). Establishing criteria for higher-level classification using molecular data: The systematics of *Polyommatus* blue butterflies (Lepidoptera, Lycaenidae). Cladistics.

[5] Gapon D.A., Kuznetsova V.G., Maryańska-Nadachowska A. (2021). A new species of the genus *Rhaphidosoma* Amyot et Serville, 1843 (*Heteroptera, Reduviidae*), with data on its chromosome complement. Comp. Cytogenet..

[6] Hebert P.D.N., Cywinska A., Ball S.L., deWaard J.R. (2003). Biological identifications through DNA barcodes. Proc. R. Soc. B Biol. Sci..

[7] Hebert P.D.N., Penton E.H., Burns J.M., Janzen D.H., Hallwachs W. (2004). Ten species in one: DNA barcoding reveals cryptic species in the neotropical skipper butterfly *Astraptes fulgerator*. Proc. Nat. Acad. Sci. USA.

[8] Talavera G., Lukhtanov V.A., Pierce N., Vila R. (2022). DNA barcodes combined with multilocus data of representative taxa can generate reliable higher-level phylogenies. Syst. Biol..

[9] Hu S.J., Condamine F.L., Monastyrskii A.L., Cotton A.M. (2019). A new species of the *Graphium* (*Pazala*) *mandarinus* group from Central Vietnam (Lepidoptera: Papilionidae). Zootaxa.

[10] Xu Z.-B., Wang Y.-Y., Condamine F.L., Cotton A.M., Hu S.-J. (2020). Are the yellow and red marked club-tail *Losaria coon* the same species?. Insects.

[11] Hu P., Lu L., Hu S., Da W., Huang C.-L., Zhang H., Wang D., Zhang Y., Xu Y., Wang R. (2022). Differentiation of the chestnut tiger butterfly *Parantica sita* (Lepidoptera: Nymphalidae: Danainae) in China. Front. Ecol. Evol..

[12] Cotton A.M., Doleck T., Zhang X., Inayoshi Y., Lohman D.J., Hu S.J. (2022). *Graphium septentrionicolus* Page & Treadaway, 2013 (Lepidoptera: Papilionidae) is a distinct species. Zootaxa.

[13] Ge S.X., Jiang Z.H., Wang J.Q., Song K., Zhang C., Hu S.J. (2023). A revision of the *Pieris napi*-complex (Lepidoptera: Pieridae) and similar species with distribution in China. Arthropod Syst. Phylogeny.

[14] Hou Y., Cao C., Chiba H., Chang Z., Huang S., Zhu L., Kunte K., Huang Z., Wang M., Fan X. (2023). Molecular phylogeny, historical biogeography, and classification of *Pseudocoladenia* butterflies (Lepidoptera: Hesperiidae). Mol. Phylogenet. Evol..

[15] Cong Q., Shen J., Zhang J., Li W., Kinch L.N., Calhoun J.V., Warren A.D., Grishin N.V. (2021). Genomics reveals the origins of historical specimens. Mol. Biol. Evol..

[16] Lemmon A.R., Brown J.M., Stanger-Hall K., Lemmon E.M. (2009). The effect of ambiguous data on phylogenetic estimates obtained by maximum likelihood and Bayesian inference. Syst. Biol..

[17] Kandul N.P., Lukhtanov V.A., Dantchenko A.V., Coleman J.W.S., Sekercioglu C.H., Haig D., Pierce N.E. (2004). Phylogeny of *Agrodiaetus* Hübner 1822 (Lepidoptera: Lycaenidae) inferred from mtDNA sequences of *COI* and *COII* and nuclear sequences of *EF1-α*: Karyotype diversification and species radiation. Syst. Biol..

[18] Kandul N.P., Lukhtanov V.A., Pierce N.E. (2007). Karyotypic diversity and speciation in *Agrodiaetus* butterflies. Evolution.

[19] Eckweiler W., Bozano G.C., Bozano G.C. (2016). Guide to the Butterflies of the Palearctic Region. Lycaenidae Part IV.

[20] de Lesse H. (1960). Les nombres de chromosomes dans la classification du groupe d’*Agrodiaetus ripartii* Freyer (Lepidoptera, Lycaenidae). Rev. Fran. Entomol..

[21] de Lesse H. (1960). Spéciation et variation chromosomique chez les Lépidoptères Rhopaloceres. Ann. Sci. Nat. Zool. Biol. Anim..

[22] Eckweiler W., Häuser C.L. (1997). An illustrated checklist of *Agrodiaetus* Hübner, 1822, a subgenus of *Polyommatus* Latreille, 1804 (Lepidoptera: Lycaenidae). Nachr. Entomol. Ver. Apollo.

[23] Olivier A., Puplesiene J., van der Pooten D., De Prins W., Wiemers M. (1999). Revision of some taxa of *Polyommatus* (*Agrodiaetus*) *transcaspicus* group with description of a new species from Central Anatolia (Lepidoptera, Lycaenidae). Phegea.

[24] Carbonell F. (2000). Contribution a la conaissance du genre *Agrodiaetus* Hübner (1822), *A. barmifiruze* n. sp. et *A. musa esfahensis* n. ssp., en Iran méridional. Linneana Belg..

[25] Carbonell F. (2001). Contribution a la connaissance du genre *Agrodiaetus* Hübner (1822), *A. ahmadi* et *A. khorasanensis* nouvelles espèces dans le Nord de l’Iran (Lepidoptera, Lycaenidae). Linneana Belg..

[26] Skala P. (2001). New taxa of the subgenus *Agrodiaetus* Hübner, 1822 from Iran: *Polyommatus* (*Agrodiaetus*) *faramarzii* sp. n., *P*. (*A*.) *shahrami* sp. n. and *P*. (*A*.) *pfeifferi astyages* ssp. n. (Lepidoptera, Lycaenidae). Nachr. Entomol. Ver. Apollo.

[27] Wiemers M. (2003). Chromosome Differentiation and the Radiation of the Butterfly Subgenus Agrodiaetus (Lepidoptera: Lycaenidae: Polyommatus)—A Molecular Phylogenetic Approach. Ph.D. Dissertation.

[28] Schurian K.G., ten Hagen W. (2003). *Polyommatus* (*Agrodiaetus*) *urmiaensis* sp. n. aus Nordwestiran (Lepidoptera: Lycaenidae). Nachr. Entomol. Ver. Apollo.

[29] Lukhtanov V.A., Kandul N.P., Plotkin J.B., Dantchenko A.V., Haig D., Pierce N.E. (2005). Reinforcement of pre-zygotic isolation and karyotype evolution in *Agrodiaetus* butterflies. Nature.

[30] Wiemers M., Keller A., Wolf M. (2009). *ITS2* secondary structure improves phylogeny estimation in a radiation of blue butterflies of the subgenus *Agrodiaetus* (Lepidoptera: Lycaenidae: *Polyommatus*). BMC Evol. Biol..

[31] Vodolazhsky D.I., Yakovlev R., Stradomsky B. (2011). Study of taxonomic status of some specimens of subgenus *Agrodiaetus* (Lepidoptera: Lycaenidae: *Polyommatus*) from Western Mongolia based on mtDNA markers. Caucas. Entomol. Bull..

[32] Lukhtanov V.A., Shapoval N.A., Dantchenko A.V. (2014). Taxonomic position of several enigmatic *Polyommatus* (*Agrodiaetus*) species (Lepidoptera, Lycaenidae) from Central and Eastern Iran: Insights from molecular and chromosomal data. Comp. Cytogenet..

[33] Rougerie R., Smith M.A., Fernandez-Triana J., Lopez-Vaamonde C., Ratnasingham S., Hebert P.D. (2011). Molecular analysis of parasitoid linkages (MAPL): Gut contents of adult parasitoid wasps reveal larval host. Mol. Ecol..

[34] Folmer O., Black M., Hoeh W., Lutz R., Vrijenhoek R. (1994). DNA primers for amplification of mitochondrial *Cytochrome c oxidase subunit I* from diverse metazoan invertebrates. Mol. Mar. Biol. Biotech..

[35] deWaard J.R., Ivanova N.V., Hajibabaei M., Hebert P.D.N., Martin C.C. (2008). Assembling DNA barcodes: Analytical protocols. Environmental Genomics, Methods in Molecular Biology.

[36] Hall T. (2011). BioEdit: An important software for molecular biology. GERF Bull. Biosci..

[37] Darriba D., Taboada G.L., Doallo R., Posada D. (2012). jModelTest 2: More models, new heuristics and parallel computing. Nat. Methods.

[38] Ronquist F., Teslenko M., van der Mark P., Ayres D.L., Darling A., Höhna S., Huelsenbeck J.P. (2012). MrBayes 3.2: Efficient Bayesian phylogenetic inference and model choice across a large model space. Syst. Biol..

[39] Balint Z. (1997). Reformation of the *Polyommatus* section with a taxonomic and biogeographic overview (Lepidoptera, Lycaenidae, Polyommatini). Neue Entomol. Nachricht..

[40] Coutsis J.G. (1985). Notes concerning the taxonomic status of *Agrodiaetus tankeri* de Lesse (Lepidoptera: Lycaenidae). Nota Lepidopterol..

[41] Coutsis J.G. (1986). The blue butterflies of the genus *Agrodiaetus* Hübner (Lep., Lycaenidae): Symptoms of taxonomic confusion. Nota Lepidopterol..

[42] Munguira M.L., Martin L., Perez-Valiente M. (1995). Karyology and distribution as tools in the taxonomy of Iberian *Agrodiaetus* butterflies. Nota Lepidopterol..

[43] Kandul N.P. (1997). The karyology and the taxonomy of the blue butterflies of the genus *Agrodiaetus* Hubner, (1822) from the Crimea. Atalanta.

[44] Wiemers M., De Prins J. (2004). *Polyommatus* (*Agrodiaetus*) *paulae* sp. nov. from Northwest Iran, discovered by means of molecular, karyological and morphological methods. Entomol. Z..

[45] Kolev Z. (2005). *Polyommatus dantchenkoi* (Lukhtanov & Wiemers, 2003) tentatively identified as new to Europe, with a description of a new taxon from the Balkan Peninsula. Nota Lepidopterol..

[46] Hesselbarth G., Van Oorschot H., Wagener S. (1995). Die Tagfalter der Türkei.

[47] Koçak A.O., Kemal M. (2001). A study on the biodiversity, zoogeography and taxonomy of the section *Agrodiaetus* Hbn. in the genus *Polyommatus* Latr. (Lycaenidae, Lepidoptera). Centre Entomol. Stud. Misc. Pap..

[48] International Commission on Zoological Nomenclature (1999). International Code of Zoological Nomenclature.

