# Supplementary material for: Phylogenetic Structure Revealed through Combining DNA Barcodes with Multi-Gene Data for Agrodiaetus Blue Butterflies (Lepidoptera, Lycaenidae)
Source: Insects. 2023 Sep 15;14(9):769. doi: 10.3390/insects14090769 (PMC10532069; doi:10.3390/insects14090769)
Supplement: Supplementary file 1 [file insects-14-00769-s001.zip › Supplementary Material 2. The command blocks.pdf]

Supplementary Material 2. The command blocks used for the Bayesian analysis of the first, second and third data sets.

We used the following command block for the first data set:

```
begin mrbayes;
	set autoclose=yes nowarn=yes;
	lset nst=6 rates=invgamma;
	unlink statefreq=(all) revmat=(all) shape=(all) pinvar=(all);
	prset applyto=(all) ratepr=variable;
	mcmcp ngen= 20000000 relburnin=yes burninfrac=0.05 printfreq=20000  samplefreq=20000 nchains=4 savebrlens=yes;
	mcmc;
	sumt;
end;
```

We used the following command block for the second data set:

```
begin mrbayes;
	charset COI = 1-1539;
	charset tRNA = 1540-1604;
	charset COII = 1605-2281;
	partition Names = 3: COI, tRNA, COII;
end;
begin mrbayes;
	set partition=Names;
	lset applyto=(1) nst=6 rates=invgamma;
	lset applyto=(2) nst=2 rates=equal;
	lset applyto=(3) nst=6 rates=invgamma;
	unlink shape=(all) pinvar=(all) statefreq=(all) revmat=(all);
	prset applyto=(all) ratepr=variable;
	mcmcp ngen= 20000000 relburnin=yes burninfrac=0.05 printfreq=20000  samplefreq=20000 nchains=4 savebrlens=yes;
	mcmc;
	sumt;
end;
```

We used the following command block for the third data set:

```
begin mrbayes;
	charset COI = 1-1539;
	charset tRNA = 1540-1604;
	charset COII = 1605-2281;
	charset ITS2 = 2282-2948;
	charset gaps=2949-2971;
	partition Names = 5: COI, tRNA, COII, ITS2, gaps;
end;
begin mrbayes;
	set partition=Names;
	lset applyto=(1) nst=6 rates=invgamma;
	lset applyto=(2) nst=2 rates=equal;
	lset applyto=(3) nst=6 rates=invgamma;
	lset applyto=(4) nst=6 rates=equal;
	unlink shape=(all) pinvar=(all) statefreq=(all) revmat=(all);
	prset applyto=(all) ratepr=variable;
	mcmcp ngen= 20000000 relburnin=yes burninfrac=0.05 printfreq=20000  samplefreq=20000 nchains=4 savebrlens=yes;
	mcmc;
	sumt;
```

end;
